# Three-Dimensional Porous Nitrogen-Doped NiO Nanostructures as Highly Sensitive NO_2_ Sensors

**DOI:** 10.3390/nano7100313

**Published:** 2017-10-11

**Authors:** Van Hoang Luan, Huynh Ngoc Tien, Seung Hyun Hur, Jong Hun Han, Wonoh Lee

**Affiliations:** 1 School of Mechanical Engineering, Chonnam National University, 77 Yongbong-ro, Buk-gu, Gwangju 61186, Korea; vhluan1986@gmail.com; 2 Department of Chemical Engineering, University of South Carolina, 301 Main Street, Columbia, SC 29208, USA; huynhngoctien@gmail.com; 3 School of Chemical Engineering and Bioengineering, University of Ulsan, 93 Daehak-ro, Nam-gu, Ulsan 44610, Korea; shhur@mail.ulsan.ac.kr; 4 School of Chemical Engineering, Chonnam National University, 77 Yongbong-ro, Buk-gu, Gwangju 61186, Korea; jhhan@jnu.ac.kr

**Keywords:** sensor, porous nanostructure, nickel oxide, nitrogen doping, NO_2_ gas sensor

## Abstract

Nickel oxide has been widely used in chemical sensing applications, because it has an excellent p-type semiconducting property with high chemical stability. Here, we present a novel technique of fabricating three-dimensional porous nitrogen-doped nickel oxide nanosheets as a highly sensitive NO_2_ sensor. The elaborate nanostructure was prepared by a simple and effective hydrothermal synthesis method. Subsequently, nitrogen doping was achieved by thermal treatment with ammonia gas. When the p-type dopant, i.e., nitrogen atoms, was introduced in the three-dimensional nanostructures, the nickel-oxide-nanosheet-based sensor showed considerable NO_2_ sensing ability with two-fold higher responsivity and sensitivity compared to non-doped nickel-oxide-based sensors.

## 1. Introduction

Nitrogen dioxide (NO_2_) is an important air pollutant because it contributes to the formation of photochemical smog, which has significantly harmful impacts on human health. Public awareness about the dangers of NO_2_ has increased the demand for detecting this hazardous gas with high precision sensors composed of highly sensitive materials. Therefore, numerous approaches have been suggested to detect NO_2_ at the ppm level [1,2,3]. Among the various types of gas sensors, semiconducting metal oxide gas sensors have attracted much attention because of their low cost, ease of production, simplicity of use, and ability to detect numerous gases, e.g., those using tungsten trioxide, titanium dioxide, vanadium oxide, tin dioxide, and nickel oxide (NiO) [4,5,6].

NiO, a well-known stable p-type semiconducting metal oxide, has a high melting point (1960 °C) and excellent chemical stability. Therefore, it has been extensively employed as electrodes in lithium ion batteries and supercapacitors, as electro-catalysts in combustion gas sensing, and in electrochromic devices [7,8,9]. Furthermore, a novel nanostructure with a high specific surface area is essential for enhanced performance of the NiO-based devices, which can be achieved by reactive sputtering, sol–gel methods, and hydrothermal treatments [10,11,12,13,14]. Therefore, highly porous NiO nanostructures can be used to fabricate highly sensitive gas sensors, especially for NO_2_ detection, because the p-type semiconducting NiO can effectively donate electrons to the NO_2_ molecules.

Previous studies have reported that nitrogen (N) is a good dopant in many metal oxides because N and oxygen have similar ionic radii [15,16,17]. N-doping in titanium dioxide effectively improved its electro-catalytic performance in the carbon monoxide gas reaction [18], and promoted NO_2_ adsorption as proven theoretically via density functional theory calculation [19]. Furthermore, the intrinsic electrical resistance and signal noise level of metal oxides could be overcome via N-doping [20,21,22].

**Figure 1:**
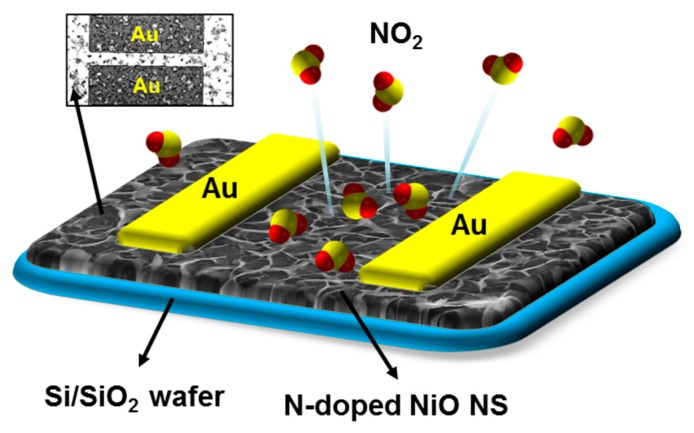
A schematic illustration and a top-view image of a three-dimensional porous N-doped NO_2_ gas sensor based on NiO nanosheets (NSs).

Herein, we present three-dimensional porous N-doped NiO nanosheets (NSs) for highly sensitive NO_2_ sensing applications. The elaborate NiO nanostructure was prepared by a simple and effective solution-based hydrothermal method [23,24]. Subsequently, N-doping was achieved by thermal treatment with ammonia gas. Figure 1 shows the N-doped NiO nanostructure used in NO_2_ sensing. The N-doped NiO nanostructure is hydrothermally grown on a silicon/silicon dioxide (Si/SiO_2_) wafer and gold (Au) electrodes are deposited to measure the electrical signals during NO_2_ exposure. Owing to the N-doping effect with hierarchical nanostructure, the resistance change in the gas sensor can be greatly amplified. The result shows that the N-doped NiO-based gas sensor, compared to a non-doped sensor, exhibits a two-fold higher responsivity and sensitivity toward NO_2_ gas detection and is thus a candidate for highly sensitive NO_2_ sensors.

## 2. Materials and Methods

### 2.1. Synthesis of the NiO Nanostructure

The three-dimensional porous NiO nanostructure was synthesized via sequential seeding and growth [23,24], and all reagents were purchased from Sigma-Aldrich, Korea. Nickel acetate tetrahydrate (0.2 M) was dissolved in a mixture of 2-methoxyethanol (6 mL) and diethanolamine (0.25 mL) to obtain an NiO seed solution. After the seed solution was stirred for 2 h at room temperature, it was spin-coated at 4000 rpm for 30 s on an Si/SiO_2_ wafer and dried on a hot-plate at 250 °C for 15 s. After the spin-coating process was repeated three times, the sample was annealed at 400 °C for 2 h in a vacuum chamber. Note that a higher rotating speed and a longer spinning time may produce an irregular and/or thin coating layer owing to excessive centrifugal force. Here, we found that the above condition was optimal for constructing uniform and stable seed layer with strong adhesion on the substrate [23]. At the next stage, an NiO growth solution was prepared by dissolving nickel nitrate hexahydrate (5.8 g) and hexamethylenetetramine (4.8 g) in distilled (DI) water (100 mL). The NiO-seed-coated wafer was dipped in the growth solution at 90 °C for 2 h, rinsed with DI water, and annealed at 350 °C for 1 h. The annealing process was performed in a gas chamber filled with argon (1 atm) and ammonia (50 sccm) for N-doping. Au electrodes were then deposited on the surface of the N-doped NiO nanostructure via thermal evaporation in vacuum.

### 2.2. Chracterization of the N-Doped NiO Nanostructure

The porous morphology of the synthesized NiO nanostructure was investigated by scanning electron microscopy (SEM, JEOL JSM-6500FE, Tokyo, Japan). X-ray diffraction (XRD) measurements were carried out to identify the crystal structure of NiO using a high-resolution X-ray diffractometer (Rigaku, D/MAXZ-2500V, Tokyo, Japan). The Brunauer–Emmett–Teller (BET) specific surface areas of the powders were determined by nitrogen adsorption in a Micromeritics ASAP 2020 (Norcross, GA, USA) nitrogen adsorption apparatus. Successful N-doping was examined by X-ray photoelectron spectroscopy (XPS) using a Thermo Fisher Scientific ESCALAB 250Xi (Waltham, MA, USA). The gas sensing properties were evaluated by measuring the resistance change with a Hewlett-Packard 4155A semiconductor parametric analyzer in a MST-5000 gas chamber (MS-Tech, Hwasung, Gyeonggi-do, Korea). In order to measure the electrical resistance, an Au layer consisting of electrodes was deposited on the surface of the N-doped NiO nanostructure using a thermal evaporator with a patterned mask. The thickness of the Au coat was 50 nm from the top surface of the NiO nanostructure and had a 10 μm spacing. The concentration of NO_2_ was carefully controlled using a mass flow controller (GMC 1200, ATOVAC, Yongin, Gyeonggi-do, Korea).

## 3. Results and Discussion

### 3.1. Morphology of the N-Doped NiO-NS Nanostructure

**Figure 2:**
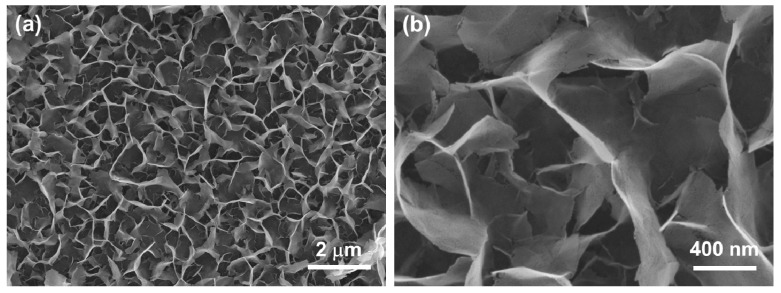
SEM images of (**a**) the three-dimensional nanostructure of N-doped NiO NSs and (**b**) its magnified image.

**Figure 3:**
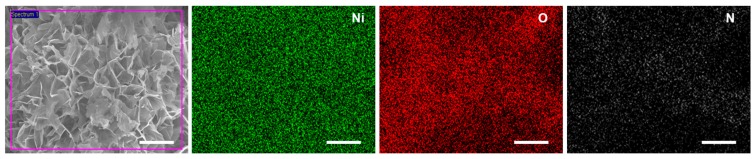
EDS mapping images of N-doped NiO NSs (scale bar = 4 μm).

The three-dimensional porous nanostructure of N-doped NiO NSs (Figure 2) shows that the thickness and height of the grown NSs were 20–30 nm and 1–3 μm, respectively, in close agreement with the previously reported values [23,24]. The porous nanostructure hierarchically grown on the wafer had a high surface area of ~205 m^2^/g, suggesting an enhanced gas adsorption ability, and can thus be applied in high-performance NO_2_ sensors. Energy-dispersive X-ray spectrometry (EDS) mapping analysis of the elements is shown in Figure 3. Uniformly dispersed green and red dots on the N-doped NiO NSs indicate the successful growth of NiO nanosheets. Note that the N-mapping in the EDS spectrum was somewhat unclear owing to the strong intensity of Ni and O. The identification of the N element was examined via XPS analysis.

### 3.2. Crystallinity of the N-Doped NiO NS

**Figure 4:**
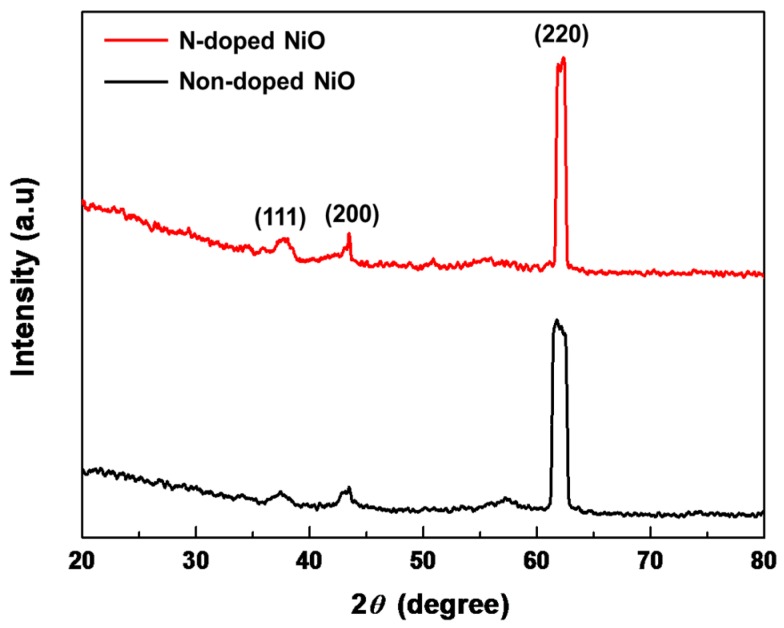
XRD results of N-doped and non-doped NiO NSs.

Figure 4 shows three distinguishable diffraction peaks at 37.2°, 43.5°, and 62.3°, corresponding to the (111), (200), and (220) NiO planes, respectively, indicating high crystallinity for both N-doped and non-doped NiO NSs [7,20,24]. All reflections in the XRD pattern can be indexed to face-centered-cubic-phase NiO (JCPDS card #47-1049) [25]. Since both peak positions and intensities are almost the same for N-doped and non-doped NiO NSs, it can be concluded that doping by nitrogen atoms has no influence on the intrinsic crystal structure of NiO. The high peak intensity indicates that the prepared NiO NSs are highly crystalline. Moreover, for the N-doped NiO NSs, crystallinity higher than that of the non-doped NiO NSs can also be verified by calculating the grain sizes from crystallographic parameters. The Scherrer’s formula was used to calculate the crystallite size *D* of the manufactured NiO NSs using Equation (1) [26,27]:

(1) $$D=\frac{0.9\lambda }{\beta \cos\theta }$$

where *λ* is the wavelength of the X-ray source (1.54 Å), *β* is the full width at half maximum of high-intensity peaks, and *θ* is the Bragg’s angle [28,29]. The obtained *β* and *D* values are listed in Table 1, where the N-doped NiO NSs have relatively larger grain sizes than those of the non-doped NSs. Therefore, it can be estimated that N-doping can increase the crystallinity of NiO NSs. The Scherrer equation is generally applicable for spherical particles; it has limitations because many factors may contribute to peak broadening in the XRD patterns. For example, the deposition and growth of materials on substrates may produce crystallographic strain due to residual stress. Herein, assuming the same circumstance for N-doped and non-doped NiO NSs, we investigated only their relative crystallinities.

### 3.3. Chemical Characterization of the N-Doped NiO NSs

**Figure 5:**
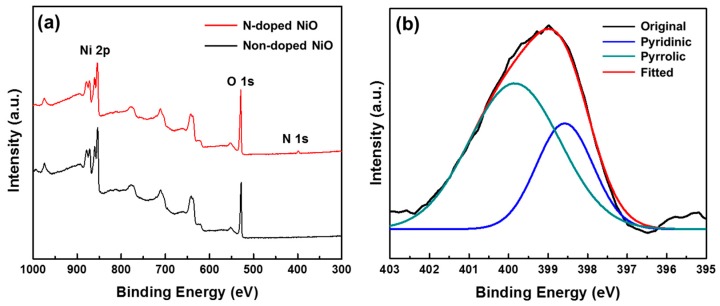
XPS spectra of N-doped and non-doped NiO NSs: (**a**) wide scan spectra and (**b**) N 1s spectra of N-doped NiO with deconvoluted peaks.

The chemical structure of NiO was examined, and N-doping was confirmed using XPS analyses. Both N-doped and non-doped NiO NSs showed the peaks of O 1s and Ni 2p (Figure 5), with two split peaks of 2p1/2 (872.1 eV) and 2p3/2 (853.2 eV), and a satellite peak of 2p3/2 (860.1 eV) [30,31]. A peak of N 1s was only observed in N-doped NiO, which indicates the existence of nitrogen atoms in N-doped NiO (Figure 5a). The high-resolution N 1s peak of N-doped NiO was deconvoluted into two peaks corresponding to pyridinic and pyrrolic nitrogen components (398.5 and 400.1 eV, respectively) [32,33]. The presence of these nitrogen species confirms N-doping on the surface of NiO NSs.

### 3.4. NO_2_ Sensing Performance of the N-Doped NiO NSs Nanostructure

**Figure 6:**
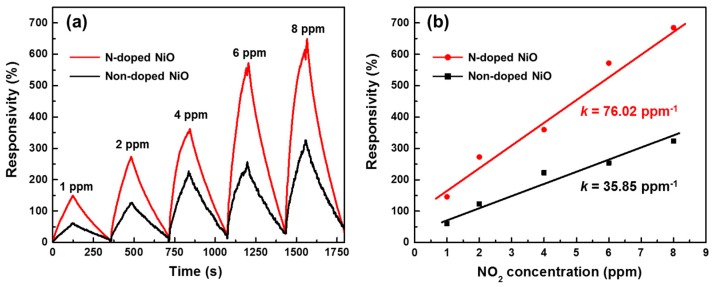
NO_2_ sensing performance of N-doped and non-doped NiO NSs: (**a**) responsivity to various gas concentrations and (**b**) sensitivity plots.

In order to elucidate the NO_2_ sensing performance of the synthesized NiO nanostructures, gas sensing tests were performed at 200 °C by exposing to various concentrations of NO_2_, from 1 to 8 ppm. Here, the exposing time was 125 s. NO_2_ was injected into the measurement chamber (200 °C) with N_2_. For a quantitative examination, the responsivity (*RS*) and sensitivity (*k*) were investigated with respect to the gas concentration, as defined in Equations (2) and (3):

(2) $$RS\left(\%\right)=\frac{R_{\mathrm{atm}}-R_{G}}{R_{\mathrm{atm}}}\times 100$$

(3) $$k\left({\mathrm{ppm}}^{-1}\right)=\frac{RS_{\max}}{C_{G}}$$

where *R*_atm_ is the resistance of the sensors measured under air circumstance and *R*_G_ is the resistance when NO_2_ gas is injected with the carrier gas. The value of *k* was obtained from the slope of the best linear fitting line in the plots of maximum responsivity (*RS*_max_) versus NO_2_ concentration (*C*_G_). Note that *R*_atm_ was measured as 13.5 kΩ. Figure 6a shows that *RS* increased when the sensor was exposed to NO_2_ because *R*_G_ decreases due to the p-type semiconducting characteristic of NiO, where the Ni^2+^ vacancies contribute to the hole-conduction [34]. After NO_2_ disposal, *RS* was reinstated to its initial stage condition, which represents reversible adsorption and desorption of NO_2_ on the surface of NiO. The N-doped NiO-NS-based sensor exhibited a two-fold higher *RS* than that of the non-doped device, which can also be confirmed by its two-fold higher *k* value in Figure 6b. Thus, there is a linear relationship between responsivity and NO_2_ concentration in both types of sensors. Hence, the NiO-NS-based device can be used as quantitative high-precision gas sensors to measure NO_2_ concentrations.

Note that other reducing gases such as H_2_S, NH_3_, and H_2_ are also applicable to N-doped NiO NS gas sensors. Because the sensing behaviors of these gases show negative responsivities with increasing values of *R*_G_ [24], selective sensing with the reducing gases and NO_2_ is possible when the NiO-NS-based gas sensors. However, the absolute values of responsivities for such reducing gases were still low (less than 50%). Therefore, it is further required to develop an innovative n-type semiconducting material, which is highly sensitive toward the reducing gases.

Even though NO can be considered for use with this gas sensor, direct use is limited because NO reacts well with O_2_ and produces NO_2_ easily under a working temperature of 200 °C. In this case, we kept the working temperature at 200 °C when degassing NO_2_ after sensing in order to remove residual and adsorbed NO_2_. Even though the measurement was performed by co-injecting N_2_ gas, the circumstances under vacuum were not ideal. Therefore, NO can be reacted with residual O_2_ at this high working temperature, and the precise amount of NO_2_ then becomes hard to control.

### 3.5. N-Doping Effect on the NiO NSs

The enhanced NO_2_ sensing performance of the N-doped NiO device is because of the increase in p-type conductivity (p^+^-type) and the decrease in material resistance by nitrogen doping. Generally, in non-doped NiO, electrical conduction occurs via the hole hopping of Ni^2+^ vacancies, where each vacancy contributes two holes for conduction associated with a p-type semiconducting characteristic. Moreover, oxygen vacancies act as shallow donors, which are responsible for n-type conductivity. Therefore, the NO_2_ molecules adsorbed to the NiO NS surfaces (NO_2_ (ads)) accept electrons from the NiO surface, and this results in the generation of holes (h^+^) as per Equation (4):

(4) $$\begin{array}{l} {\mathrm{NO}}_{2}\left(\mathrm{gas}\right)\to {\mathrm{NO}}_{2}\left(\mathrm{ads}\right)\\ {\mathrm{NO}}_{2}\left(\mathrm{ads}\right)\to {\mathrm{NO}}_{2}^{-}\left(\mathrm{ads}\right)+h^{+}. \end{array}$$

When NO_2_ is adsorbed on the N-doped NiO surface, holes are generated on the surface by an electron transfer mechanism to the NO_2_ molecules. Then, the holes on the N-doped NiO surfaces are transferred to the non-doped NiO surface (p-type). This hole-transfer at the p^+^–p heterojunction leads to a decrease in resistance of the NiO backbone, which carries charges between the two electrodes [35,36]. Without such heterojunctions, the generated holes are likely to accumulate on the NiO surface, causing relatively low responsivity owing to the suppression of further adsorption of NO_2_ molecules. Therefore, the p^+^–p heterojunctions can relieve the amassed holes and can promote the continuous adsorption of NO_2_, which results in the high responsivity and sensitivity of the sensors. Therefore, the synergetic effect of the p-type semiconducting property of NiO and the p-type doping of N increased the performance of the NO_2_ sensing.

### 3.6. Repetitive and Saturate Responses of the N-Doped NiO-NSs-Based NO_2_ Sensor

**Figure 7:**
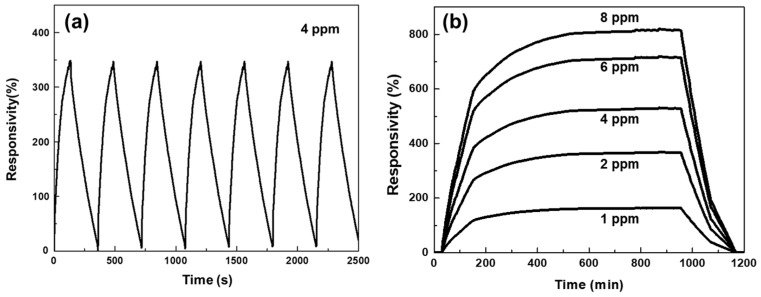
(**a**) Repetitive and (**b**) saturate responses of the N-doped NiO-NS-based NO_2_ sensors.

Furthermore, in order to examine the stability of the N-doped NiO-nased gas sensor, a repetitive NO_2_ gas sensing test was performed at a specific NO_2_ gas concentration of 4 ppm. The NO_2_ gas flow was controlled by sequentially turning on (125 s) and turning off (250 s) and the test was done at 200 °C. As shown in Figure 7a, the synthesized gas sensor exhibited stable reversibility of the gas sensing characteristics under repetitive NO_2_ exposure conditions, supporting its potential application in NO_2_ sensors, where cycle-to-cycle variation can be successfully removed. For every cycle, the N-doped NiO-based sensor showed highly amplified responsivity (resistance change) upon NO_2_ exposure and fully recovered signals in the absence of NO_2_ with good repetitive switching behaviors. With high sensitivity, highly stable, and repetitive gas sensing performance was observed, which suggests that the presented N-doped NiO nanostructure-based device has great potential for applications in quantitatively reliable sensors to precisely measure NO_2_ gas.

Additionally, the N-doped NiO-based sensor showed stable saturate responses for all different concentrations of NO_2_, as shown in Figure 7b. After certain injection times, the values of responsivities exhibited steady-state behaviors, which confirms that the developed sensor can give reproducible responses for detecting NO_2_. For the saturate response, the value of *k*_sat_ (Equation (3)) was calculated as 90.13 ppm^−1^ by linear-fitting (root mean square = 0.96). This result shows that N-doped NiO-NS-based gas sensors are highly sensitive toward NO_2_ gas. Note that previous work using the non-doped NiO NS on a graphene substrate showed *k* = 32.4 ppm^−1^. Because the measurement circumstance was kept at a working temperature of 200 °C, even after the gas sensing by NO_2_ injection, all residual and adsorbed gases could be fully removed from the NiO NSs. Therefore, the device can exhibit stable responses for long-term cycle. It is noted that the N-doped NiO-based gas sensor showed negligible change on repetitive and saturate responses after one month.

## 4. Conclusions

NiO has been widely used in chemical sensing applications, because it has an excellent p-type semiconducting property and high chemical stability. Here, in order to develop highly sensitive and stable NO_2_ sensors, we successfully fabricated three-dimensional porous N-doped NiO NSs by a simple and effective hydrothermal technique. N-doping of this novel NiO NS led to significantly enhanced NO_2_ sensing performance with a two-fold higher responsivity and sensitivity compared to non-doped NiO NS nanostructure. This was achieved by constructing p^+^–p heterojunctions at the N-doped NiO sites. The novel N-doped NiO nanostructure-based device exhibited high sensitivity and stability with good repetitive gas sensing performance. Hence, it is a candidate of choice for quantitatively reliable high-precision gas sensors for the measurement of NO_2_ gas.

## Figures and Tables

**Table 1:** Structural crystal parameters of N-doped and non-doped NiO NSs.

Sample	*β* (Deg)	*D* (nm)
N-doped NiO	0.65 ± 0.02	26.3 ± 0.8
Non-doped NiO	1.42 ± 0.05	12.0 ± 0.5
